# Influence of Biotreatment on *Hordeum vulgare* L. Cereal Wholemeal Contamination and Enzymatic Activities

**DOI:** 10.3390/foods12051050

**Published:** 2023-03-01

**Authors:** Grazina Juodeikiene, Karolina Trakselyte-Rupsiene, Karolina Reikertaite, Elizabet Janic Hajnal, Vadims Bartkevics, Iveta Pugajeva, Valentas Gruzauskas, Mantas Švazas, Romas Gruzauskas, Antonello Santini, João Miguel Rocha, Elena Bartkiene

**Affiliations:** 1 Department of Food Science and Technology, Kaunas University of Technology, LT-50254 Kaunas, Lithuania; 2 Institute of Food Technology, University of Novi Sad, 21000 Novi Sad, Serbia; 3 Faculty of Chemistry, University of Latvia, LV-1004 Riga, Latvia; 4 Institute of Food Safety, Animal Health and Environment BIOR, LV-1076 Riga, Latvia; 5 Institute of Computer Science, Vilnius University, LT-08303 Vilnius, Lithuania; 6 Department of Pharmacy, University of Napoli Federico II, 80131 Napoli, Italy; 7 Laboratory for Process Engineering, Environment, Biotechnology and Energy (LEPABE), Faculty of Engineering, University of Porto (FEUP), 4200-465 Porto, Portugal; 8 Associate Laboratory in Chemical Engineering (ALiCE), Faculty of Engineering, University of Porto (FEUP), 4200-465 Porto, Portugal; 9 Institute of Animal Rearing Technologies, Faculty of Animal Sciences, Lithuanian University of Health Sciences, LT-44307 Kaunas, Lithuania; 10 Department of Food Safety and Quality, Faculty of Veterinary, Lithuanian University of Health Sciences, LT-44307 Kaunas, Lithuania

**Keywords:** biological detoxification, barley, lactic acid bacteria, trichothecenes, deoxynivalenol, deoxynivalenol conjugates

## Abstract

Crop contamination with mycotoxins is a global problem with a negative impact on human and animal health as well as causing economical losses in food and feed chains. This study was focused on the evaluation of the effect of lactic acid bacteria (LAB) strain (*Levilactobacillus brevis*-LUHS173, *Liquorilactobacillus uvarum*-LUHS245, *Lactiplantibacillus plantarum*-LUHS135, *Lacticaseibacillus paracasei*-LUHS244 and *Lacticaseibacillus casei*-LUHS210) fermentation on the changes in the level of deoxynivalenol (DON) and its conjugates in *Fusarium* spp.-contaminated barley wholemeal (BWP). Samples, with different contamination of DON and its conjugates, were treated separately (for 48 h). In addition to mycotoxin content, enzymatic activities (amylolytic, xylanolytic, and proteolytic) of BWP (before and after fermentation) were evaluated. It was established that the effect of decontamination depends on the LAB strain used, and a significant reduction in DON and the concentration of its conjugates in *Lc. casei* fermented samples was achieved: the amount of DON decreased on average by 47%, and the amount of D3G, 15-ADON and 3-ADON decreased by 82.4, 46.1, and 55.0%, respectively. *Lc. casei* also showed viability in the contaminated fermentation medium and an effective production of organic acids was obtained. Additionally, it was found that enzymes are involved to the detoxification mechanism of DON and its conjugates in BWP. These findings indicate that fermentation with selected LAB strains could be applied for contaminated barley treatment in order to significantly reduce *Fusarium* spp. mycotoxin levels in BWP and improve the sustainability of grain production.

## 1. Introduction

Barley is the world’s fourth most widely grown cereal grain, and is popular in temperate zones, including North-western Europe and Canada. Approximately 140 million tons of barley is produced annually in the world, and is mainly used for feed (70%) and brewing (27%) [1]. According to the production volume, this cereal remains among the most important raw materials, so it is important to ensure its microbiological safety. Due to uncontrolled climatic conditions (temperature or relative air humidity), microscopic fungi that can release secondary metabolites—mycotoxins—can appear in barley grains as well as in other plant products such as grapes [2].

It is known that barley is considered less sensitive to mycotoxins due to the different composition of its anatomical parts compared to wheat, but *Fusarium* spp. fungi signs of infection in barley grains are much more difficult to identify than in wheat [1]. A lot of research is being conducted to evaluate mycotoxins in wheat grains or to solve their detoxification possibilities, but similar research related to the prevention of mycotoxin contamination should also be carried out during barley processing [1].

*Fusarium* spp. microscopic fungi-produced mycotoxin deoxynivalenol (DON) is among the most common representatives of the trichothecene group in grain products, especially in European climate conditions. This mycotoxin is not degraded during food production because it is chemically resistant and thermally stable. Trichothecens, such as DON, are toxic to both humans and animals. Mycotoxins affect the imune system, induce vomiting, affect weight gain and have other negative symptoms [3]. In organisms, during metabolism, DON transformations are possible by switching to conjugated (masked) forms. Such chemical modifications are aimed at reducing the initial toxicity of mycotoxins by forming compounds with lower toxicity. These DON conjugates are also toxic and can cause adverse effects, but they have been little studied and regulated, e.g., those proposed are not yet not available. It is important to mention that the appearance of mycotoxins in crops, e.g., in barley, not only has a negative impact on human health but also negatively affects production processes in the food industry. In the brewing industry, barley contamination causes foaming in drinks, and possibly even bottle explosions.

Currently, the European Union (EU) only regulates DON by setting the maximum allowed concentrations of this mycotoxin: 1250 μg/kg—in unprocessed cereals, 200–750 μg/kg—in cereal products for human consumption, and 200 μg/kg—in cereal-based baby food [4]. DON conjugates are still not regulated. In 2011, the United Food and Agriculture Organization (FAO), the World Health Organization (WHO), and the Expert Committee on Food Additives (JECFA) addressed acetylated DON conjugates (3-ADON and 15-ADON) by establishing a provisional maximum daily allowance for body weight (1 μg/kg) [5]. The EU Food Safety Authority (EFSA) has recently been active in assessing the risks associated with conjugated mycotoxins and establishing health-related reference values for individual groups of *Fusarium* mycotoxins together and their conjugated forms. Both institutions clearly emphasized the need to urgently continue the work on the identification of yet uncharacterized conjugated mycotoxins, gathering more data on the occurrence of known modified mycotoxins in food and feed and studying their toxic kinetics and toxicity. According to the EFSA, in Europe, 15 to 55% of barley products are contaminated with DON [6] and 2 to 50% with T-2/HT-2 [7]. Detectable concentrations of DON were reported: ~484 μg/kg in unprocessed barley; 152 μg/kg in barley grain intended for human consumption; 8.4–11.3 μg/kg in beer; and 187 μg/kg in feed [5,8]. D3G is commonly found in barley, wheat, corn, and oats, as well as in grain products (bread, beer, and malt) [9]. Published data show that nearly 30% of grain was contaminated with D3G and free toxins [10]. 3-ADON and 15-ADON are also commonly found in cereal grains and their products (breakfast, fiber-enriched bread, oatmeal, etc.) [11].

Therefore, it is important to look for methods which would allow reducing not only DON but also DON conjugates, thus detoxifying grain raw material, as this problem is relevant all over the world, including Europe. There are various ways to prevent mycotoxin contamination but more and more attention is being paid to environmentally friendly biocontrol, such as fermentation with lactic acid bacteria (LAB). LAB have antimicrobial properties, including antifungal activity, and are generally recognized as safe (GRAS) [12].

According to Magnusson [13], the antimicrobial degradation mechanism of LAB can be explained by the fact that they accumulate organic acids and produce antifungal compounds, which are antagonistic to pathogens. Among the most studied LAB strains, *Lactiplantibacillus plantarum* can synthesize peptides or antimicrobial proteins known as bacteriocins. Among the samples with the highest antifungal activity, *Lp. plantarum* produced DL-3-phenyllactic acid, salicylic acid and vanillin, and the same compounds were also produced by the other tested *Lp. plantarum* strain. These compounds synthesized by *Lp. plantarum* are known to induce cell perturbations and stress, resulting in plasma membrane lysis [14]. Other references suggest that enzymes such as epoxidases can break down the toxic ring of trichothecenes. Therefore, the detoxification of DON may also be related to the action of oxidative enzymes catalyzing the breakdown of the epoxy ring present in the DON structure [15].

In the present research study, the efficiency of barley biomass bioprocessing has been explored, and the peculiarities of biochemical processes in *Fusarium* spp. and contaminated grains during LAB fermentation have also been studied.

## 2. Materials and Methods

### 2.1. Barley Grain Samples and Wholemeal Preparation

Different *Fusarium* spp.-contaminated samples of barley grain were obtained from the northern part of Serbia (Vojvodina province) in 2019 and were stored in plastic bags in a freezer at −18 °C. Barley grain samples were grounded using a laboratory mill (Bühler-Miag, Brunswick, Germany) and the obtained barley wholemeal products (indicated as BWP1 to BWP5) were further used for fermentation.

Barley wholemeal (BWP) samples for the analysis of mycotoxins (μg/kg) and their fermentation were prepared at the KTU Department of Food Science and Technology. Prepared BWP were tested at the Latvian Food Safety, Animal Health and Environmental Research Institute BIOR.

### 2.2. Lactic Acid Bacteria

Five strains of LAB were used for the fermentation of BWP—*Levilactobacillus brevis* LUHS173, *Liquorilactobacillus uvarum* LUHS245, *Lactiplantibacillus plantarum* LUHS135, *Lacticaseibacillus paracasei* LUHS244 and *Lacticaseibacillus casei* LUHS210—which were isolated from spontaneous sourdough and provided from the collection of the Lithuanian University of Health Sciences [16]. Prior the use, LAB strains were maintained at −80 °C in a 25% glycerol solution in a Microbank system (Pro-Lab Diagnostics, Merseyside, UK) and before the fermentation strains were activated in MRS broth (de Man, Rogosa, and Sharpe, CM 0359, Oxoid Ltd., Hampshire, UK) for 48 h at 30 °C.

### 2.3. Biotreatment of Barley Wholemeal Product Samples

For fermentation, each sample of BWP (15 g) was mixed with sterile distilled water (30 mL) to reach 65% moisture. Then, 1.35 mL (3% of the total weight) of the corresponding LAB culture was used for sample innoculation [17]. Inoculated BWP samples were incubated anaerobically in a TC160 thermostat (SalvisLab Thermocenter, Vernon Hills, IL, USA) for up to 48 h at 30 °C. During fermentation, samples were collected at different stages (after 0, 24 and 48 h) for the evaluation of LAB viable counts and pH measurement.

The determination of LAB viable counts in BWP was performed according to Bartkiene et al. [18]. Decimal dilutions of the sample with sterile saline solution (9 g/L) were prepared and inoculated into Petri dishes with sterile MRS agar using the spread plate technique. Samples were incubated under anaerobic conditions at 30 °C for 72 h. All results were expressed in log_10_ CFU/mL.

After fermentation, the remaining fermented BWP samples were lyophilized and stored under dark and dry conditions prior analysis of mycotoxins by HPLC-TOF-HRMS. Initial barley samples, contaminated by *Fusarium* spp., were used as a control (non-fermented samples). During the fermentation process, the pH was determined using a pH meter (PP-15; Sartorius, Göttingen, Germany).

### 2.4. Methods of Analysis

#### 2.4.1. High-Performance Liquid Chromatography

Mycotoxin analysis (µg/kg) was performed using a high-performance liquid chromatography (HPLC) system (UltiMate 3000, Thermo Fisher Scientific, Waltham, MA, USA) coupled to a compact Q-TOF time-of-flight high-resolution mass spectrometer (Bruker, Bremen, Germany) according the sample preparation and analysis procedure conditions as described previously [12]. For sample preparation, a modified QuEChERS method, described by Reinholds et al. [12], was used.

Chromatographic separation was carried out with a Phenomenex Kinetex C_18_ reversed-phase analytical column (1.7 μm, 100 Å, 50 × 3.00 mm; USA) at a flow-rate of 0.35 mL/min. A positive full scan mode for analysis of all mycotoxins over the m/z scanning range from 50 to 1000 was used. The mass extraction window applied for quantification purposes was set to ±5 ppm at 10,000 full-width half-maximum (FWHM) resolution. For control of data processing a HyStar 3.2 software (Bruker Daltonik GmbH, Bremen, Germany) was used, and data analization was performed using a QuantAnalysis 4.3 software (Bruker Daltonik GmbH, Bremen, Germany).

Analyzed mycotoxins in BWP samples were as follows: DON (LOD-4.0 µg/kg; LOQ-12 µg/kg), D3G (LOD-4.5 µg/kg; LOQ-13 µg/kg), 3-ADON (LOD-3.6 µg/kg; LOQ-11 µg/kg) and 15-ADON (LOD-14.0 µg/kg; LOQ-42.0 µg/kg) [19].

#### 2.4.2. Evaluation of Enzymatic Activities in Barley Wholemeal Product Samples

Enzymatic profiles, which could be related to both fungi and LAB growth, of BWP samples were determined using the sample preparation and analysis procedure conditions previously reported [17]. Amylolytic activity was evaluated according to ICC Standard Method 108 [20]. Under the action of α-amylase, soluble starch is hydrolyzed to dextrins of various molecular weights, and remaining unhydrolyzed starch content is determined by the colorimetric reaction with iodine and the absorbance was read at 670 nm.

Xylanolytic activity was determined using the 3,5-dinitrosalicylic acid (DNS) method [21]. Xylanase hydrolyzes xylan to reducing saccharides, mainly xyloses, which forms a coloured compound with DNS in a strongly alkaline environment and the absorbance was read at a wavelenght of 540 nm.

Determination of proteolytic activity was performed according to Cupp-Enyard [22] using 0.65% (*w/v*) casein as substrate. Under the action of protease, casein is broken down into tyrosine and other amino acids. Folin and Ciocalteu’s phenolic reagent reacts with free tyrosine and produces a blue-colored chromophore, which is quantifiable and measured as an absorbance value of the 660 nm.

For the spectrophotometric analysis, a spectrophotometer Genesys 10UV (Thermo Fischer Scientific, Waltham, MA, USA) was used to measure changes in optical density of the samples.

### 2.5. Statistical Analysis

All analyzes were performed in triplicate, and results are presented as the mean values (n = 3) ± standard error (SE). Statistical analysis was performed using statistical package SPSS for Windows [v28.0.1.0 (142), SPSS, Chicago, IL, USA]. The Shapiro–Wilk test was used to indicate normality of the data. To evaluate the effects of LAB fermentation to reduce DON levels in BWP, a multivariate analysis of variance (ANOVA) with Tukey’s test was applied and results were considered as statistically significant at *p* ≤ 0.05. A linear Pearson’s correlation was determined to quantify the strength of the correlation between the variables.

## 3. Results and Discussion

### 3.1. Characterization of DON and Its Conjugated Forms in Barley Wholemeal Products

The most common trichothecenes—DON and its conjugates (D3G, 3-ADON, and 15-ADON)—have been analyzed using the HPLC-TOF-HRMS method in a collection of BWP (BWP1-BWP5) and obtained results are presented in Table 1. The results confirmed the presence of DON in BWP together with its modified forms. DON is the primary mycotoxin which occurs in the plant as a metabolite of fungi and DON conjugates are derivatives of DON [23]. Most of the BWP samples with lower contamination of DON showed significantly lower levels of conjugated DON forms, compared to its free form. The most analyzed modified mycotoxin is D3G, which is a phase II metabolite of DON, and it is recognized that D3G is less toxic with respect to DON [24]. D3G was detected in barley together with DON, its primary form. Moreover, high concentrations of D3G have been detected in beer [25,26].

According to the literature [27], human colonic microbiota can convert D3G back to DON. To date, the level of D3G in cereals and their products is not regulated. Therefore, since D3G can be converted back to DON and reactivate its toxicity, also the D3G/DON ratio should be considered [3]. In this study, the D3G/DON ratio in the evaluated BWP samples varied from 21.09% to 69.35%. The obtained results agree with the work of other authors, showing that the ratio of D3G to DON can varied from 20 to 70% [28]. Several studies have shown that D3G contamination levels in grain and grain by-products reached half the level of DON. D3G can be present together with DON in crops and grain foods, as well as in feed in variable amounts which can reach 100%, and concentrations in the range from 2 to 1700 μg/kg [29,30]. The D3G/DON ratio in grain reached 20% and exceeded 100% after treatment [30,31]. The D3G/DON ratio varied among BWP samples depending on the DON content. The results show that the barley samples (BWP1-BWP4) with lower DON content (248–996 μg/kg) were characterized by a higher D3G/DON ratio (45.65% on average) than the sample (BWP5) with the highest DON concentration (6563 μg/kg), where the D3G/DON ratio averaged 27.12%.

These results are in agreement with Pinton et al. [32], who reported that D3G content is related to DON level, and the samples with the lowest DON concentration showed the highest levels of D3G. Since this modified mycotoxin can be toxic for humans and animals, D3G levels should be evaluated and food and feed, safety should be ensured, paying particular attention to products with levels of contamination lower than the maximum acceptable concentration of DON.

Other DON metabolites, such as 3-ADON and 15-ADON, were identified in this work in BWP and were also detected in food and feed. These modified forms of DON are digested by intestinal microbiota and can turn into a primary toxin or can cause internal toxicity; 15-ADON localized in the body can be more toxic than DON, indeed [33]. According to the EFSA [5], the ratio of the masked acetylated DON forms (3-ADON and 15-ADON) and DON-3-glucoside (D3G) to DON is 10, 15, and 20%, respectively, and it should be noted that they concurred with observations in feed as well as in unprocessed grains of undefined end-use, except for ‘alcoholic beverages’ (‘beer and beer-like beverage’), where the ratio of D3G to DON was evaluated as 80%.

The results of our research show that DON levels in BWP may significantly exceed the permissible limit concentration of this toxin, and such cereal raw material cannot be used for food or feed production. The increased pollution of barley is possibly related to climate change, and not only it increases the loss of cereal raw materials but also poses an urgent environmental problem. The data of DON conjugates confirm relatively high amounts of D3G and 15-ADON in the tested BWP and especially their close correlation with DON. Thus, it is important to carry out strict monitoring of free trichothecenes and their glucoside conjugates and apply sustainable management measures.

### 3.2. The Effect of Fermentation on Deoxynivalenol and Its Conjugates Concentrations in Barley Wholemeal Products

Biological control methods are still under research for the reduction in *Fusairum* spp. mycotoxins in contaminated cereals. Among the alternatives is the fermentation of grains with antimicrobial LAB. To evaluate the efficiency of biological decontamination process it is important to evaluate the resistance of LAB strains to the contamination of the fermentation medium. The changes on the count of LAB and samples pH during the 48 h fermentation process in contaminated with *Fusarium* spp. BWP are presented in Table 2. Microbiological analysis of fermented samples showed that the growth intensity of LAB dependent on the contamination level of the medium. LAB strains, such as *Lc. paracasei* and *Lev. brevis*, were the most resistant and were maintained to effectively multiply in both low- and high-contamination samples, LAB count increased, on average, by 35.0 and 24.3%, respectively (Table 2). Other LAB strains, *Liq. uvarum*, *Lp. plantarum,* and *Lc. casei*, were more sensitive to the medium contamination—LAB count increased, on average, by 15.7%.

The optimal pH conditions for the growth of LAB is in between the range of 5.5–6.2, but LAB strains are resistant to acidic media and are able to multiply at pH around 4.5, and some strains can grow at even lower pH [11,34]. Normally, bacterial growth stops at pH 3.5 [34]. The slower growth of LAB strains in the contaminated medium could have negative impact for the formation of organic acids, such as lactic, sorbic, and propionic acid, which are responsible for the antimicrobial properties of LAB strains [35]. Obtained results show that all LAB strains remained active in the contaminated medium and the intensive production of organic acids was observed, reducing the pH of the medium from 5.2–6.4 at the beginning of fermentation to an average of <3.5 after 48 h of fermentation (Table 2).

**Figure 1:**
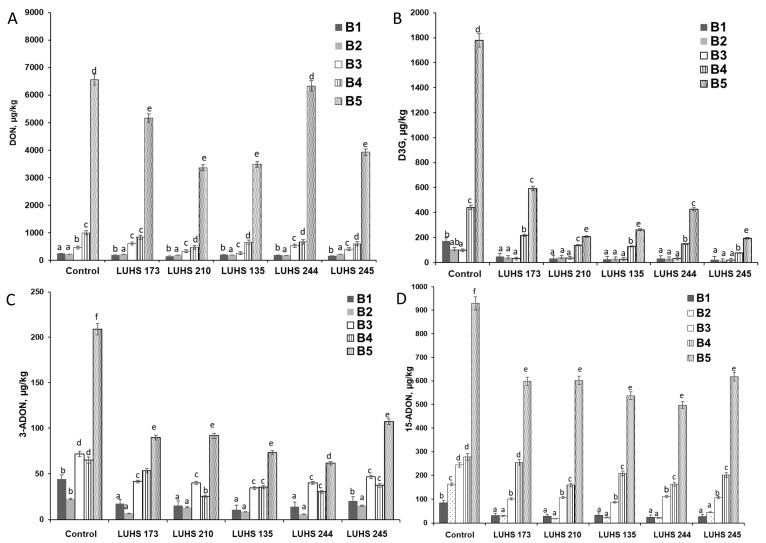
Changes in concentrations of DON (**A**) and DON conjugates [D3G (**B**), 3 –ADON (**C**), and 15-ADON (**D**)] during fermentation in barley wholemeal product samples (BWP1–BWP5) with different contamination using different LAB strains (*Levilactobacillus brevis* LUHS173, *Lacticaseibacillus casei* LUHS210, *Lactiplantibacillus plantarum* LUHS135, *Lacticaseibacillus paracasei* LUHS244, and *Liquorilactobacillus uvarum* LUHS245). DON—deoxynivalenol; D3G—deoxynivalenol-3-glucoside; 3-ADON-3-acetyldeoxynivalenol and 15-ADON-15-acetyldeoxynivalenol. ^a–f^ Mean values denoted with different letters indicate significantly different values between the columns (*p* ≤ 0.05).

At the next stage of this study, the influence of the fermentation with different LAB strains on the qualitative and quantitative composition of DON, D3G, 3-ADON, and 15-ADON, in different BWP samples contaminated with *Fusarium* spp., was evaluated (Figure 1). The results of the analysis of the DON concentration (before and after fermentation) confirmed that the degree of mycotoxin biodegradation during fermentation (expressed as the average DON content) was found to be, on average, by 31% lower than that of the control sample (Figure 1). Fermentation of the highest contaminated samples (6563 μg/kg DON) reduced DON, on average, by 32.2%, compared to biologically treated less contaminated DON samples (<1000 μg/kg). Thus, LUHS245 fermentation can achieve a significant reduction in DON at different levels of BWP contamination (6563, 996, and 248 μg/kg DON) to 1.7, 1.7 and 1.6 fold, respectively. During the fermentation with LUHS210, LUHS135, and LUHS173, DON content in highly contaminated (6563 μg/kg) fermented samples was reduced by 48.8, 46.9, and 21.3%, compared to the control sample, and with LUHS244 only by 3.6% (Figure 1A).

Quantitative analysis of conjugated forms of DON showed a significant reduction in D3G and the acetylated forms of DON (namely 3-ADON and 15-ADON) in fermented BWP samples. After fermentation, the D3G concentration was reduced to, on average, by 78.0%, with respect to the control sample (Figure 1B). The reduction in the 3-ADON and 15-ADON concentrations (on average, by 54.6 and 45.4%, respectively) was also noted in the fermented BWP samples (Figure 1C,D).

The results obtained in this experiment revealed that most of the LAB strains showed a decrease in the amount of several DON conjugates during fermentation, highlighting the influence of the LAB strain on these changes. It was reported that the level of LAB biological degradation depends on LAB cell density and viability as well as on the pH of the medium [36]. The LAB strains used in the experiment differed in these characteristics, so the observed response of the LAB strains to the level of contamination of the medium was different.

Biological detoxification of mycotoxins during fermentation is possible through several mechanisms, e.g., absorption of mycotoxins by LAB viable cells or enzymatic degradation of mycotoxins and interactions between fermentation metabolites and mycotoxins [36,37].

The obtained results agree with the results of other researchers [38], confirming the ability of different LAB strains to reduce the amount of mycotoxins such as DON and fumonisins B1 and B2. It was found that *Lactobacillus delbrüeckii* ssp. *bulgaricus* reduced DON up to 55% and *Lactobacillus rhamnosus* strain GG (LGG) reduced DON up to ~54% [38]. The potential for a reduction in other trichothecenes, such as T-2 toxin (T-2) and HT-2 toxin (HT-2), nivalenol (NIV), diacetoxyscirpenol (DAS) and fusarenon (FX), during fermentation was also investigated and described in other studies [39]. The results presented in this study confirmed that the respective LAB strains were capable of binding trichothecenes and the results show that the effectiveness of LAB in reducing trichothecenes (20 μg/mL) significantly differed between the tested mycotoxins and LAB strains. The percentage of bound mycotoxins was in the range 18–93% [38], but none of the LABs used were able to bind 3-ADON [30]. In order assure the safety of fermented barley products, the toxicity of the LAB–mycotoxin complex must be considered [30]. According to the literature [40,41,42], during biological detoxification of DON by bacteria and fungi through adsorption or enzymatic degradation, new less toxic by-products are formed, such as DOM-1, 3-keto-DON, 3-epi-DON, and 3-oxo-DON. Future research is needed to identify by-products of barley wholemeal fermentation with selected LAB strains and to evaluate their toxicity.

As referred to previously, the enzymatic activity of fungi in contaminated grain (before fermentation) may also be involved in the mechanism of biological degradation of mycotoxins converting the latters to non-toxic compounds during LAB fermentation. The enzymatic degradation of *Fusarium* spp.-producing mycotoxins during the fermentation process is less studied; for this reason, the enzymatic profiles of BWP samples will be taken into consideration and their correlation with the tested mycotoxin concentrations will be studied at the next stage of the experiment.

### 3.3. Changes in Barley Wholemeal Products Enzymatic Activities during Fermentation and Their Correlation with Mycotoxin Concentration

**Figure 2:**
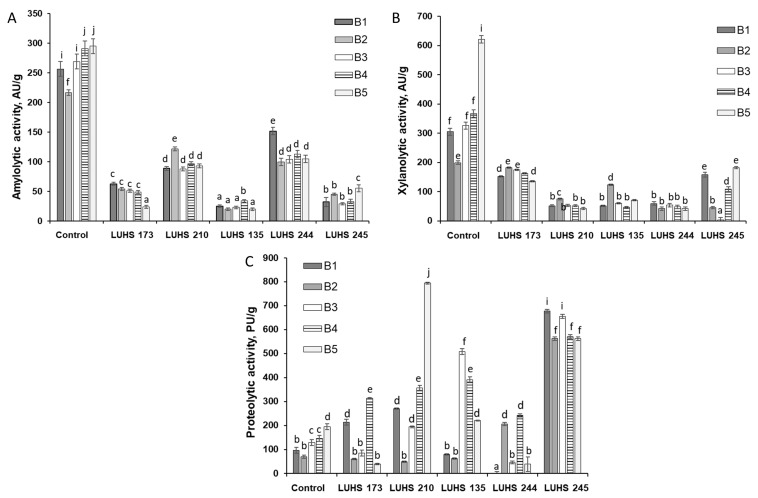
Changes in amylolytic (**A**), xylanolytic (**B**), and proteolytic (**C**) activities in barley wholemeal samples (BWP1-BWP5) during fermentation with LAB strains (AU—amylolytic and xylanolytic enzyme activity units; PU—proteolytic enzyme activity units; *Levilactobacillus brevis* LUHS173, *Lacticaseibacillus casei* LUHS210, *Lactiplantibacillus plantarum* LUHS135, *Lacticaseibacillus paracasei* LUHS244, and *Liquorilactobacillus uvarum* LUHS245). ^a–j^ Mean values denoted with different letters indicate significantly different values between the columns (*p* ≤ 0.05).

**Figure 3:**
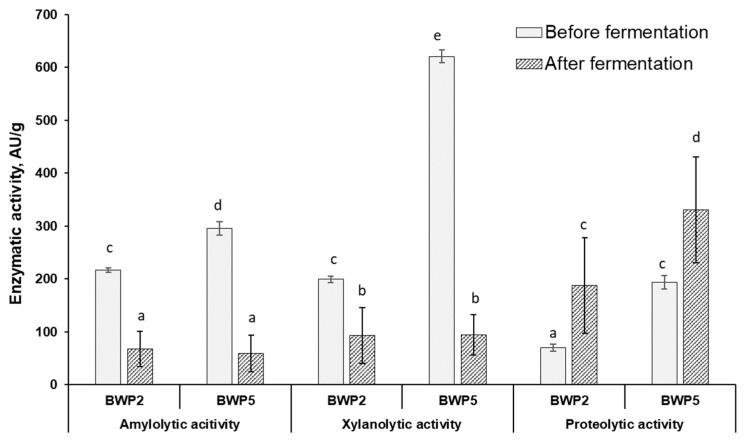
Changes in enzymatic activities (amylolytic, xylanolytic, and proteolytic) during fermentation in barley wholemeal samples with the lowest (BWP2) and highest (BWP5) contamination (AU—activity units). ^a–e^ Mean values denoted with different letters indicate significantly different values between the columns (*p* ≤ 0.05).

**Figure 4:**
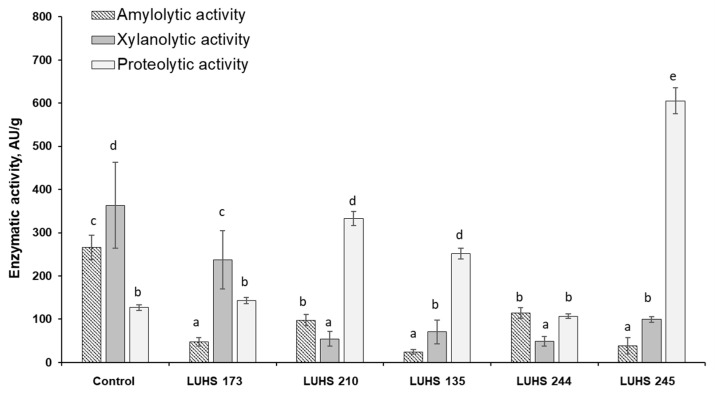
Changes in enzymatic activities (amylolytic, xylanolytic, and proteolytic) of LAB strains (LUHS 173, LUHS 210, LUHS 135, LUHS 244, LUHS 245-barley wholemeal samples fermented with *Levilactobacillus brevis* LUHS173, *Lacticaseibacillus casei* LUHS210, *Lactiplantibacillus plantarum* LUHS135, *Lacticaseibacillus paracasei* LUHS244, and *Liquorilactobacillus uvarum* LUHS245, respectively) during fermentation of the least contaminated sample BWP2 (AU—activity units). ^a–e^ Mean values denoted with different letters indicate significantly different values between the columns (*p* ≤ 0.05).

During this experiment, enzymatic activity profiles (amylolytic, proteolytic, and xylanolytic) and their peculiarities during LAB fermentation were analyzed. Research results are presented in Figure 2, Figure 3 and Figure 4, respectively, and correlations are given in Supplementary File S1 Table S1.

The amylolytic activity of enzymes during fermentation decreased, in comparison with the raw material before fermentation, on average from 266 to 65 AU/g (Figure 2A). Comparing the fermented BWP samples with each other in terms of amylolytic activity, a difference between the LAB strains used for fermentation is apparent: the highest average enzymatic activity was found in LUHS244 and LUHS210 fermented samples (115 and 98 AU/g, respectively), while significantly lower values of the tested enzymatic activity were attained in LUHS135, LUHS245, and LUHS173 fermented ones (on average, 24, 39 and 48 AU/g, respectively). The results of studies of xylanolytic activity during fermentation, presented in Figure 2B, show a significant decrease in their average from 364 AU/g (in raw material before fermentation) to 87 AU/g (after fermentation).

When comparing BWP fermented with different LAB strains according to xylanolytic activity values, LUHS173 and LUHS245 (some samples) have higher activity than LAB such as LUHS135, LUHS210 and LUHS244. When assessing the correlation between the xylanolytic activity of LAB strains in fermented BWP and the DON concentrations, a strong correlation was obtained only between the number of viable counts of LUHS245 and DON (r = 0.5965, *p* ≤ 0.05) and also between the number of viable counts of LUHS210 and DON (r = 0.6231 *p* ≤ 0.05). No correlations between other LAB strains and DON were detected.

The results of proteolytic activity tests during the fermentation of BWP samples, presented in Figure 2C, show an increase in activities, on average, from 127 to 288 PU/g. The highest increase in proteolytic activity, compared to other LAB strains, occurred in LUHS245 fermented samples (on average, 605 PU/g) and it did not depend on the level of contamination of the sample. A significant increase in proteolytic activity, compared to nontreated BWP, was also recorded during LUHS135 and LUHS210 fermentation, and it was more pronounced in samples with higher levels of contamination (BWP3 and BWP4).

BWP samples contaminated with *Fusarium* spp. demonstrated different enzymatic activities (amylolytic, xylanolytic, and proteolytic) and relationship between enzymatic activities and level of mycotoxins was taken into consideration (Supplementary File S1 Table S1). A very strong correlation was found between the proteolytic and xylanolytic activities of fungi and DON content (r = 0.8443, r = 0.9470, respectively, *p* ≤ 0.05), as well as with D3G, 3-ADON, and 15-ADON (r = 0.8534, r = 0.9515, with D3G; r = 0.9130, r = 0.9806 with 3-ADON; and r = 0.8737, r = 0.9411 with 15-ADON, respectively, *p* ≤ 0.05). Strong correlation between amylolytic enzyme activity in different contaminated BWP and the presence of mycotoxins (3-ADON, D3G and 15-ADON) (r = 0.7056, r = 0.6267 and r = 0.6190, respectively, *p* ≤ 0.05) was obtained, indicating that *Fusarium* toxins producing fungi also possess amylolytic activity.

When assessing the correlations between amylolytic activity in BWP fermented with different LAB strains and the DON concentration, a very strong correlation was found between the number of viable count of LUHS173 and the DON concentration (r = 0.9605 *p* ≤ 0.05), as well as between the number of viable counts of LUHS245 and DON (r = 0.8063, *p* ≤ 0.05). Conversely, no statistically significant correlations were detected between other LAB strains and DON (LUHS135- r = 0.3055, LUHS210- r = 0.2116 and LUHS244- r = 0.2841, *p* ≤ 0.05).

Additionally, the comparative results of the study of enzymatic activities (amylolytic, xylanolytic, and proteolytic) during LAB fermentation (before and after fermentation) in samples with lowest and highest DON contamination (BWP2 and BWP5, respectively) are graphically presented in Figure 3. Judging from the changes in enzymatic activities during fermentation in the less contaminated samples group (BWP2), it is possible to predict the influence of LAB enzymes on the mycotoxin detoxification process, and according to the results of the enzymatic activities of sample with the highest level of contamination (BWP5) the influence of the filamentous fungi participation in the detoxification process of the studied mycotoxins can be predicted. Figure 3 shows the average increase in the proteolytic activity of both LAB and fungi during fermentation from 70 to 188 PU/g (in sample BWP2) and from 194 to 331 PU/g (in sample BWP5), respectively.

A strong positive correlation (r = 0.7490, *p* ≤ 0.05) was found between proteolytic enzyme activity and the DON concentration in the sample with higher fungi contamination, suggesting the possible participation of fungal proteolytic enzymes in the DON detoxification mechanism. Conversely, the effect of other enzymatic activities (including xylanolytic activity) on the reduction in the tested mycotoxin concentrations was not significant.

*Fusarium* spp. survives in a wide range of temperature and pH value. According to the literature [43], isolates of *Fusarium graminearum* differ significantly in terms of temperature and pH requirements. The isolates were found to grow better under the temperature range of 25–30 °C and pH 5.0, but the growth-rate varied [43]. Some researchers found out a better growth of *Fusarium oxysporum* and *Fusarium solani* at pH 4.5 and 6.0, and *F. graminearum* and *Fusarium equise* at pH 3.5 and 6.0, respectively [43]. Thus, fungi can grow and form spores under a wide pH range, averaging from 4.0 to 8.0. Some Fusarium spp. species can grow and form spores at the pH range from 5.0 to 6.0 [43].

In this experiment, the pH values of the fermented BWP were between 3.5 and 4.0. According to the literature [43], this pH it is still possible for *Fusarium* spp. to growth. Finally, it can be assumed that the proteolytic activity of fungi was activated in the highly contaminated BWP, which could have influenced on the reducing of mycotoxin concentrations.

The increase in proteolytic activity in among the least contaminated samples (BWP2) also indicates the possible involvement of LAB in the detoxification process (Figure 4).

LUHS245 and LUHS210 stood out with the highest proteolytic activity in all BWP samples. When investigating the correlation between the proteolytic activity of LAB in fermented BWP and the DON concentration in fermented BWP samples, a very strong positive correlation was found between LUHS210 and DON (r = 0.9324, *p* ≤ 0.05) and a moderate positive correlation was recorded between LUHS245 and DON (r = 0.4655, *p* ≤ 0.05), although this strain was distinguished by high values of proteolytic activity in all samples. No significant correlations were found between other LAB strain counts and the DON concentration. It should be noted that LUHS210 also grew up well during fermentation in BWP with different contamination levels of *Fusarium* and produced organic acids. Among the most effective reductions in the DON concentration was recorded in LUHS210 fermented BWP. The novelty of this research is that biological treatment of BWP with *Lc. casei* strain proved to contribute to a very effective and statistically significant (*p* ≤ 0.05) reduction in mycotoxins (DON and its conjugated forms), which could be related to microbial conjugation of mycotoxins as well as with enzymatic degradation of mycotoxins by fungal enzymes.

Based on the obtained results, it can be seen that amylolytic enzyme activity had a strong correlation with filamentous fungi capable to forming DON and DON conjugates and this activity decreased during LAB fermentation. A very strong correlation was found between the amylolytic activity of some LAB strains and the DON concentration in fermented samples. The xylanolytic and proteolytic activities had a very strong connection with the presence of fungi, participating in the process of mycotoxin formation, in the cereal raw material. However, xylanolytic activity values decreased in most cases during fermentation, showing no correlation between separate LAB activity and mycotoxin concentrations. It can be assumed that the fungi with proteolytic activity in grain raw material can be activated in fermentation conditions and participate in combination with LAB (e.g., *Lc. casei*) in the detoxification process of mycotoxins. Thus, the possibility can not be ruled out that both filamentous fungi and LAB can act in a complex way in the detoxification process of mycotoxins, and their proteolytic activity, according to the results of this experiment, would have the most important significance for this.

## 4. Conclusions

Barley grains can be biologically decontaminated by prolonged LAB fermentation. Our results demonstrate that the concentrations of DON and its conjugates, identified in BWP samples contaminated with *Fusarium* spp., using the UHPLC-Orbitrap-HRMS technique, were significantly reduced during fermentation with selected LAB strains: DON concentration from 1698 to 1180 μg/kg on average, D3G concentration from 519.2 to 113.96 μg/kg, 3-ADON concentration from 82.4 to 37.36 μg /kg and 15-ADON concentration from 339.4 to 185.2 μg/kg. The decontamination effect depends on the LAB strain used, and the highest reduction in mycotoxins concentration was achieved by using the *Lc. casei* strain for fermentation. In particular, the DON concentration in BWP samples, considering the average value, decreased by 47%, while the D3G, 15-ADON and 3-ADON concentrations decreased by 82.4, 46.1, and 55%, respectively. Selected LAB strains showed a resistance to contamination in the medium and were maintained to effectively multiply in both low- and high-contamination samples and produced organic acids. LAB strains, such as *Lc. paracasei* and *Lev. brevis*, were the most resistant LAB counts, increased on average by 35.0 and 24.3%, respectively, while the LAB count of other strains, *Liq. uvarum*, *Lp. plantarum,* and *Lc. casei*, increased, on average, by 15.7%.

Biochemical studies of *Fusarium* spp.-contaminated BWP samples showed that during the fermentation process, a decrease in the activity of amylases and xylanases was recorded, on average, by 4 fold. In contrast, the activity of proteases increased in both (with low and high contamination) BWP samples, on average by 2.2 fold. Analysis of enzymatic activities (amylolytic, xylanolytic, and proteolytic) and their correlation with mycotoxin concentrations (before and after fermentation) showed that *Fusarium* spp.-producing DON and the tested DON conjugates exhibit a wide range of enzymatic activities. Our findings suggest that fungal enzymes could be involved in combination with LAB (*Lc. casei*) in the detoxification of mycotoxins.

## Figures and Tables

**Table 1:** Concentrations (μg/kg) of deoxynivalenol and its conjugates in barley wholemeal initial samples.

Samples	DON, µg/kg	D3G, µg/kg	D3G/DON, %	15-ADON, µg/kg	15-ADON/DON, %	3-ADON, µg/kg	3-ADON/DON, %
BWP1	248 ± 12.5 ^a^	172 ± 8.6 ^a^	69.35 ± 3.5 ^a^	84 ± 4.2 ^a^	33.87 ± 1.7 ^b^	44 ± 2.2 ^b^	17.74 ± 0.9 ^e^
BWP2	223 ± 11.2 ^a^	107 ± 5.4 ^a^	47.98 ± 2.4 ^b^	162 ± 8.1 ^b^	72.65 ± 3.6 ^d^	22 ± 1.1 ^a^	9.80 ± 0.5 ^c^
BWP3	460 ± 23.0 ^a^	97 ± 4.9 ^a^	21.09 ± 1.1 ^a^	244 ± 12.2 ^c^	53.04 ± 2.7 ^c^	72 ± 3.6 ^c^	15.65 ± 0.8 ^d^
BWP4	996 ± 49.8 ^b^	440 ± 22.0 ^b^	44.18 ± 2.2 ^b^	278 ± 13.9 ^c^	27.91 ± 1.4 ^b^	65 ± 3.3 ^c^	6.52 ± 0.3 ^b^
BWP5	6563 ± 328.2 ^c^	1780 ± 89.0 ^c^	27.12 ± 1.4 ^a^	929 ± 46.5 ^d^	14.12 ± 0.7 ^a^	209 ± 10.5 ^d^	3.18 ± 0.2 ^a^

BWP1-BWP5-barley wholemeal product samples; DON—deoxynivalenol; D3G—deoxynivalenol-3-glucoside; 3-ADON-3-acetyldeoxynivalenol and 15-ADON-15-acetyldeoxynivalenol. Results are expressed as the mean of three determinations (n = 3) ± standard error (SE). ^a–e^ Mean values denoted with different letters indicate significantly different values between the columns (*p* ≤ 0.05); Mycotoxin detection limits were as follows: DON (LOD-4.0 µg/kg; LOQ-12 µg/kg), D3G (LOD-4.5 µg/kg; LOQ-13 µg/kg), 3-ADON (LOD-3.6 µg/kg; LOQ-11 µg/kg) and 15-ADON (LOD-14.0 µg/kg; LOQ-42.0 µg/kg).

**Table 2:** Changes in pH and lactic acid bacteria (LAB) counts during 48 h fermentation of barley wholemeal (BWP) samples (BWP–BWP6).

Samples				Fermentation Duration			
		0 h		24 h		48 h	
		pH	LAB, log_10_ CFU/g	pH	LAB, log_10_ CFU/g	pH	LAB, log_10_ CFU/g
*Lev. brevis* (LUHS 173)	BWP1	6.37 ± 0.23 ^a^	7.88 ± 0.21 ^b^	3.83 ± 0.02 ^b^	9.64 ± 0.20 ^d^	3.87 ± 0.06 ^b^	9.34 ± 0.20 ^d^
	BWP2	5.93 ± 0.04 ^a^	7.33 ± 0.02 ^b^	3.90 ± 0.01 ^b^	9.45 ± 0.18 ^d^	3.99 ± 0.02 ^b^	9.36 ± 0.15 ^d^
	BWP3	5.62 ± 0.02 ^a^	7.54 ± 0.10 ^b^	3.86 ± 0.01 ^b^	9.36 ± 0.15 ^d^	3.96 ± 0.05 ^b^	9.35 ± 0.18 ^d^
	BWP4	5.60 ± 0.02 ^a^	7.51 ± 0.12 ^b^	3.85 ± 0.03 ^b^	9.15 ± 0.10 ^d^	3.94 ± 0.01 ^b^	9.38 ± 0.40 ^d^
	BWP5	5.56 ± 0.07 ^a^	7.43 ± 0.09 ^b^	3.92 ± 0.01 ^b^	9.58 ± 0.16 ^d^	3.95 ± 0.01 ^b^	9.37 ± 0.25 ^d^
*Liq. uvarum* (LUHS 245)	BWP1	5.75 ± 0.06 ^a^	7.11 ± 0.10 ^b^	3.79 ± 0.03 ^b^	9.04 ± 0.10 ^d^	3.57 ± 0.07 ^b^	8.35 ± 015 ^c^
	BWP2	5.72 ± 0.01 _a_	7.14 ± 0.12 ^b^	3.85 ± 0.01 ^b^	8.93 ± 0.09 ^cd^	3.75 ± 0.01 ^b^	8.57 ± 0.10 ^c^
	BWP3	5.50 ± 0.02 ^a^	7.43 ± 0.15 ^b^	3.86 ± 0.02 ^b^	8.46 ± 0.12 ^c^	3.75 ± 0.01 ^b^	8.42 ± 0.21 ^c^
	BWP4	5.52 ± 0.01 ^a^	7.09 ± 0.08 ^b^	3.87 ± 0.01 ^b^	8.50 ± 0.16 ^c^	3.77 ± 0.02 ^b^	8.50 ± 0.20 ^c^
	BWP5	5.24 ± 0.08 ^ab^	7.26 ± 0.12 ^b^	3.91 ± 0.02 ^b^	8.52 ± 0.20 ^c^	3.79 ± 0.03 ^b^	8.52 ± 0.15 ^c^
*Lp. plantarum* (LUHS 135)	BWP1	6.10 ± 0.05 ^a^	7.19 ± 0.20 ^b^	3.53 ± 0.02 ^b^	7.43 ± 0.15 ^b^	3.45 ± 0.01 ^b^	8.05 ± 0.19 ^bc^
	BWP2	6.15 ± 0.11 ^a^	7.07 ± 0.08 ^b^	3.55 ± 0.01 ^b^	7.29 ± 0.12 ^b^	3.48 ± 0.02 ^b^	8.08 ± 0.20 ^bc^
	BWP3	5.89 ± 0.04 ^a^	7.12 ± 0.05 ^b^	3.58 ± 0.05 ^b^	7.25 ± 0.09 ^b^	3.50 ± 0.02 ^b^	8.85 ± 0.26 ^cd^
	BWP4	5.80 ± 0.05 ^a^	7.10 ± 0.09 ^b^	3.60 ± 0.01 ^b^	7.48 ± 0.20 ^b^	3.51 ± 0.01 ^b^	7.85 ± 0.12 ^b^
	BWP5	5.81 ± 0.01 ^a^	6.66 ± 0.05 ^ab^	3.57 ± 0.01 ^b^	8.03 ± 0.13 ^bc^	3.48 ± 0.11 ^b^	7.94 ± 0.09 ^bc^
*Lc. paracasei* (LUHS 244)	BWP1	5.81 ± 0.04 ^a^	5.40 ± 0.07 ^a^	3.66 ± 0.02 ^b^	7.91 ± 0.15 ^bc^	3.52 ± 0.01 ^b^	7.42 ± 0.14 ^b^
	BWP2	5.63 ± 0.01 ^a^	5.53 ± 0.10 ^a^	3.71 ± 0.04 ^b^	7.38 ± 0.10 ^b^	3.54 ± 0.03 ^b^	7.46 ± 0.20 ^b^
	BWP3	5.59 ± 0.12 ^a^	5.47 ± 0.15 ^a^	3.72 ± 0.02 ^b^	7.52 ± 0.25 ^b^	3.53 ± 0.01 ^b^	7.36 ± 0.14 ^b^
	BWP4	5.65 ± 0.08 ^a^	5.52 ± 0.12 ^a^	3.72 ± 0.01 ^b^	7.42 ± 0.40 ^b^	3.55 ± 0.01 ^b^	7.52 ± 0.09 ^b^
	BWP5	5.56 ± 0.09 ^a^	5.79 ± 0.20 ^a^	3.73 ± 0.05 ^b^	7.41 ± 0.12 ^b^	3.56 ± 0.05 ^b^	7.64 ± 0.18 ^b^
*Lc. casei* (LUHS 210)	BWP1	6.00 ± 0.03 ^a^	7.01 ± 0.09 ^b^	3.70 ± 0.01 ^b^	8.09 ± 0.20 ^bc^	3.58 ± 0.07 ^b^	8.31 ± 0.22 ^c^
	BWP2	6.02 ± 0.03 ^a^	7.25 ± 0.10 ^b^	3.71 ± 0.01 ^b^	7.87 ± 0.20 ^b^	3.58 ± 0.01 ^b^	7.83 ± 0.09 ^b^
	BWP3	5.84 ± 0.02 ^a^	7.23 ± 0.15 ^b^	3.72 ± 0.01 ^b^	7.81 ± 0.15 ^b^	3.59 ± 0.02 ^b^	8.13 ± 0.21 ^bc^
	BWP4	5.90 ± 0.03 ^a^	7.12 ± 0.09 ^b^	3.71 ± 0.02 ^b^	7.72 ± 0.20 ^b^	3.60 ± 0.01 ^b^	8.02 ± 0.19 ^bc^
	BWP5	5.66 ± 0.01 ^a^	7.16 ± 0.20 ^b^	3.71 ± 0.05 ^b^	7.73 ± 0.18 ^b^	3.59 ± 0.01 ^b^	8.27 ± 0.23 ^c^

BWP—barley wholemeal products; LAB—lactic acid bacteria; CFU—colony forming units. Results are expressed as the mean of three determinations (n = 3) ± standard error (SE). ^a–d^ Mean values denoted with different letters indicate significantly different values between the columns (*p* ≤ 0.05).

## Data Availability

All datasets generated for this study are included in this article.

## Supplementary Materials

The following are available online at https://www.mdpi.com/article/10.3390/foods12051050/s1, Supplementary File S1. Table S1. Relationship between mycotoxin levels and enzymatic activities of tested barley samples.
