# Supplementary material for: Influence of Biotreatment on Hordeum vulgare L. Cereal Wholemeal Contamination and Enzymatic Activities
Source: Foods. 2023 Mar 1;12(5):1050. doi: 10.3390/foods12051050 (PMC10001146; doi:10.3390/foods12051050)
Supplement: Supplementary file 1 [file foods-12-01050-s001.zip › foods-2218148-supplementary.pdf]

Supplementary Table S1. Relationship between mycotoxin levels and enzymatic activities of tested barley samples.

Samples		Correlations between tested parameters, *r*		
		**Amylolytic activity**	**Xylanolytic activity**	**Proteolytic activity**
Control	DON	0.5962	0.9470	0.8443
	D3G	0.6267	0.9515	0.8534
	3-ADON	0.7056	0.9806	0.8737
	15-ADON	0.6190	0.9411	0.9130
LUHS 173	DON	0.9607	0.4892	0.4286
LUHS 210	DON	0.2116	0.6173	0.9324
LUHS 135	DON	0.3055	0.0616	0.9333
LUHS 244	DON	0.2841	0.5222	0.3232
LUHS 245	DON	0.8063	0.5967	0.4672

Levilactobacillus brevis LUHS173, *Lacticaseibacillus casei* LUHS210, *Lactiplantibacillus plantarum* LUHS135, *Lacticaseibacillus paracasei* LUHS244 and *Liquorilactobacillus uvarum* LUHS245; DON – deoxynivalenol; D3G - deoxynivalenol-3-glucoside; 3-ADON - 3-acetyldeoxynivalenol and 15-ADON - 15-acetyldeoxynivalenol.
